# Immune-related protein signature in serum stratify relapsed mantle cell lymphoma patients based on risk

**DOI:** 10.1186/s12885-020-07678-4

**Published:** 2020-12-07

**Authors:** Lavanya Lokhande, Venera Kuci Emruli, Arne Kolstad, Martin Hutchings, Riikka Räty, Mats Jerkeman, Sara Ek

**Affiliations:** 1 Department of Immunotechnology, Lund University, Lund, Sweden; 2 Oslo University Hospital, Oslo, Norway; 3 Department of Haematology, Rigshospitalet, Copenhagen, Denmark; 4 Department of Hematology, Helsinki University Central Hospital, Helsinki, Finland; 5 Department of Oncology, Lund University, Lund, Sweden

**Keywords:** Mantle cell lymphoma (MCL), Serum proteins, Biomarker discovery, Protein signature

## Abstract

**Background:**

Response to modern treatment strategies, which combine cytotoxic compounds with immune stimulatory agents and targeted treatment is highly variable among MCL patients. Thus, providing prognostic and predictive markers for risk adapted therapy is warranted and molecular information that can help in patient stratification is a necessity. In relapsed MCL, biopsies are rarely available and molecular information from tumor tissue is often lacking. Today, the main tool to access risk is the MCL international prognostic index (MIPI), which does not include detailed biological information of relevance for different treatment options. To enable continuous monitoring of patients, non-invasive companion diagnostic tools are needed which can further reduce cost and patient distress and enable efficient measurements of biological markers.

**Methods:**

We have assessed if serum-based protein profiling can identify immune related proteins that stratify relapsed MCL patients based on risk. Overall, 371 scFv targeting 158 proteins were assessed using an antibody microarray platform. We profiled patients (*n* = 44) who had been treated within the MCL6-Philemon trial combining targeted and immune-modulatory treatment.

**Results:**

The downstream processing led to the identification of the relapsed immune signature (RIS) consisting of 11 proteins with potential to stratify patients with long and short overall survival (OS). Moreover, in this population, MIPI alone failed to separate high, intermediate and low risk patients, but a combined index based on MIPI together with RIS, MIPI_ris_, showed improved performance and significantly stratified all three risk groups based on OS.

**Conclusions:**

Our results show that addition of biological parameters to previous prognostic indices improves patient stratification among patients treated with BTK inhibitor triplet combination, particularly, in the identification of an extreme high risk group.

**Supplementary Information:**

The online version contains supplementary material available at 10.1186/s12885-020-07678-4.

## Background

An effective anti-tumor immune response plays a major role for the outcome of cancer treatment [1]. This is exemplified by the success of immuno-oncology drugs [2] and the prognostic impact of immune cells, such as T-cells and macrophages in a wide range of cancer sub-types [3, 4]. Thus, methods to assess immune profiles in patients are in high demand.

To be able to select the optimal treatment for a patient, novel efforts to use information from liquid biopsies are of major interest, as they represent a minimally invasive method that can mirror the systemic immune response to the tumor and also contain circulatory proteins and DNA secreted from the tumor itself [5]. They allow the possibility to follow the patient status over time, thus monitoring temporal variation of treatment response.

Additionally, diagnostic/prognostic tests need to be robust to be useful for their intended specific group. Unfortunately, single biomarkers often have low accuracy. Thus, the approach of utilising signatures over single biomarkers for gaining diagnostic and prognostic information, has successfully been employed in a number of studies [6–15]. The rationale is that a combination would provide far more mechanistic information, higher discriminatory power and improved biological insight [16]. Thus, the use of signatures in therapeutic decision making, development of companion diagnostic tools and personalized medicine is being explored.

In this study, we have combined three important concepts of immune focused analysis; minimally invasive sampling to evaluate if combination of proteins detected in serum can be used to risk stratify patients diagnosed with relapsed mantle cell lymphoma (MCL). The goal is to define a minimally invasive method that can help stratify patients and allow treatment selection in order to optimize outcome and reduce treatment related side-effects.

The current golden standard in the clinic today for prognostication in MCL is the mantle cell lymphoma index, MIPI [17], which was developed for diagnostic patients and its applicability in relapsed patients is unclear. In general, the number of scientific studies focusing on relapsed MCL patients and prognostic information is limited. Few risk factors have been studied and are limited to Ki-67 and MIPI [18, 19]. The lack of tumor material from relapsed/refractory patients is a limiting factor for studies of molecular characteristics associated with relapse and refractory disease. Although it is not known how much the biology changes for the individual patient during the course of the disease, it is hypothesized that additional molecular factors can contribute to outcome. Today, when a wide range of treatment options are available in the relapsed/refractory setting, improved information on molecular features and related risk in relapsed patients is important. It is also essential that clinical tools for decision making are developed based on information gathered in homogenously treated cohorts of patients, to understand the relationship between molecular features and outcome in relapsed/refractory patients.

To identify protein signatures correlated to survival and improve patient stratification based on immune and clinicopathological parameters, we have profiled serum protein markers in a cohort of relapsed MCL patients previously collected during the Philemon trial conducted by the Nordic Lymphoma group [19]. In that trial, relapsed patients were treated with lenalidomide, rituximab and ibrutinib based on R2 induction therapy, as previously described in Ruan et al [20]. Proteins were detected using the affinity-based proteomic platform IMMray [13, 14, 21–24], which allowed us to analyze 371 proteins in serum samples collected prior to treatment initiation. Downstream analysis led to the identification of an 11 protein signature, which in combination with MIPI, robustly separate patients based on risk. The new index, referred to as MIPI_ris_ (relapsed immune signature combined with MIPI) could significantly differentiate the high-risk patient subgroup and improve the overall patient stratification in this relapsed MCL patient cohort compared to either MIPI or the immune signature alone.

## Methods

### Patient cohort

Relapsed/refractory (R/R) MCL patient serum samples were collected from the phase two MCL6-Philemon trial (NCT02460276) (https://clinicaltrials.gov/ct2/show/NCT02460276) conducted by the Nordic Lymphoma group. The study period was 2015–2018. Samples were collected from ten clinical sites within Sweden, Denmark, FInland and Norway. Patient eligibility criteria included age > 18 years, at least one previous regimen with rituximab and measurable site of disease [19]. Patients were treated with an induction phase of 12 cycles (28 days each) with rituximab (1400 mg subcutaneously or 375 mg/m2 intravenously; once during week one and then once every eighth week), ibrutinib (orally, 560 mg daily) and lenalidomide (orally 15 mg per day, 1–21 in each cycle). This was followed by a maintenance phase (56 days) where the patients were given ibrutinib and rituximab only. Patients were enrolled at ten clinical sites in the Nordic countries during April 2015 and June 2016, and were followed for two years. The overall treatment response rate was evaluated using PET and CT. Overall survival (OS) was calculated as the time from study enrolment to the date of death/last follow-up; whereas progression free survival (PFS) was calculated as the time from study enrolment to date of disease progression/last follow-up/death [19]. The relapsed samples included in the study were collected at the time of enrolment in the clinical trial, prior to initiation of any treatment cycle and with a minimum of 30 days since last front-line therapy. Serum samples were stored at − 80 °C until the day of the experiment.

### Labelling of serum samples

Serum samples were biotinylated using previously optimized protocols. In brief, serum samples were first diluted (1:5 in 1XPBS) and placed on an orbital shaker at 300 rpm, 4 °C for 10 min. The samples were then labelled with equal volume of 2.56 mM of Biotin solution (EZ-link Sulfo-NHS-LC-Biotin (Pierce, Rockford, IL, USA)) for two hours at 4 °C on an orbital shaker. Tris-HCL (0.5 mM) was used for termination of the biotinylation reaction, for 20mins at 4 °C on an orbital shaker. Along the labelling process, three replicates of a reference serum sample were included as process control. The biotinylated samples were finally aliquoted and stored at -80 °C until further analysis.

### Production of human recombinant antibodies

In total, 371 human recombinant His-tagged single chain variable fragments (scFv) targeting 158 immunoregulatory and tumor-associated serum proteins (Supplementary Table 1), were produced and harvested in *E. coli*, and purified using MagneHis protein purification system (Promega, Madison, WI) and Zeba 96-well desalt spin plates (Thermo Fisher Scientific), according to manufacturer’s protocol. Nanodrop quantification and SDS-Page was used to measure the protein yield and purity respectively.

The specificity, affinity, and on-chip functionality of the scFv’s has been assured using stringent phage display selection protocols [25, 26], multiple clones (one to nine) per target, and a molecular design adapted for microarray application [27]. In addition, the specificity of several of the antibodies has previously been validated using well-characterized human samples and multiple orthogonal methods [22, 23, 26, 28].

### Detection of serum proteins using antibody microarrays

The purified scFv fragments were further printed on black polymer MaxiSorp microarray slides (NUNC, Roskilde, Denmark) using a non-contact printer SciFlexarrayer S11 (Scienion, Berlin, Germany). Two columns, each containing seven identical subarrays were printed on each microarray slide. Each scFv analyte was printed in three replicates within each subarray (Supplementary Figure S1). BSA-biotin and PBS were used as positive and negative controls, respectively. A total of seven samples could be analyzed on one single slide.

The entire protocol has been previously optimized and standardized [13, 21, 24]. Briefly, each slide was mounted in individual hybridization gaskets. Slides were blocked for 1 h with constant shaking, using a blocking solution of 1% (w/v) milk in PBST (1% v/v Tween20 in 1XPBS) and washed with four cycles of PBST (Tween20 in 1XPBS, 0.05% v/v). Biotinylated serum samples, diluted 1:50 in blocking solution were then added onto the slides and incubated for 2 h with constant agitation, to allow the serum proteins to conjugate to their respective scFv fragments. Six serum samples and one quality control sample was added to each slide. The slides were again washed four times with PBST and then incubated for 1 h with 1 μg/ml of Streptavidin tagged with Alexa Fluoro 647 (Invitrogen). After the last washing step, the slides were immersed in distilled water and quickly dried with a stream of nitrogen gas and scanned immediately using Innoscan 710 (Innopsys, France) at 635 nm.

### Antibody microarray data pre-processing

To quantify spots and evaluate signal intensities, the IMMray Evaluation Software (IES, Immunovia AB, Lund, Sweden) was used. Primarily, each scanned subarray was carefully assessed for their overall quality and signal quantification. For any defects detected (background variation, uneven spots, slide scratches, spot leakage etc.) that affected any spot, the spot was removed from the downstream analysis. If > 30% of the spots in a subarray were discarded due to poor quality, the assay for that particular sample was repeated. Finally, local background was removed, and signals were extracted as an average over three replicates when the cumulative variation (CV) was below 15%. If the CV was above 15% the outlier replicate was eliminated, and the final signal represented the average of the remaining two replicates. In addition, all mean signals were trimmed, meaning that 5% of the lower and upper extreme values were discarded.

For the initial analysis, the raw signals were log2-transformed and potential batch variations were assessed. Analysis and visualization was performed using three dimensional principal component analysis (PCA) with variance filtering and hierarchical clustering on Qlucore Omics Explorer (Qlucore, Lund, Sweden) and orthogonal partial least square supervised clustering on SIMCA 15 (Umetrics, Sartorius). Several technical and clinical parameters were tested for batch effect identification. Scan date (three array days) and slide batch (two slide batches printed on different days but using the same set of antibody production) were shown to cause batch effects, with scan date being the dominant factor. ComBat normalization which uses empirical Bayes framework [29] using R (Surrogate variable analysis (SVA) package, www.r-project.org) with scan date as a covariate was used to remove the batch effect.

### Antibody microarray data analysis

Two parallel regression methodologies were employed to minimize false positive analytes being identified, as no validation cohort was available. In the first approach, the prognostic relevance (OS) in relation to each protein analyzed was evaluated by univariate cox regression analysis. The applicability of the cox regression model was validated by testing the independence between scaled Schoenfeld residuals with respect to time. A non-significant correlation for all parameters certified the validity of the proportionality hazard assumption. The list of biomarkers identified through cox regression underwent a secondary step for further variable reduction using stepwise backward elimination algorithm complemented with support vector machine and leave-one-out cross validation, using receiving operator characteristics (ROC) as the error metric. The full process, from now on referred to as Cox-BE, is described in detail in Supplementary Materials and Methods.

The second approach utilized elastic net regression (ENR) to identify key prognostic (OS) proteins. The dataset was randomly split between training (80%) and test (20%) groups. Multiple models were developed with varying elastic net mixing parameter (α∈[0, 1] with increments of 0.1; for ridge regression α = 0 and for lasso regression α = 1). Each model performance was estimated by the root mean square error (RMSE) and R^2^ values. The model with the least RMSE and highest R^2^ value was selected. Elastic net regression shrinks the coefficient to zero for redundant variables, thus, reducing the variable list providing a condensed panel of proteins associated with OS. The final signature (*n* = 11) was selected based on the overlap in proteins identified using the two separate methods. A summary of the experimental and bioinformatic pipeline is shown in Supplementary Figure S2.

### Analysis of condensed protein signature and development of combined risk score

Pathway analysis (STRING: The Search Tool for the Retrieval of Interacting Genes/Proteins, (http://stringdb.org) was used to analyse the protein interaction and functional overlap between the serum proteins defining the signature. Additionally, the signature panel was used to define a protein signature score (Supplementary materials and methods S2.) based on the predicted regression coefficients from the univariate cox analysis. The signature score was determined in a way that enabled partially categorical, ternary division of the dataset (*n* = 44) (Supplementary materials and methods S2). This signature score was further used to define the new MIPI_ris_ index (Supplementary materials and method S2.). To categorize the dataset into various risk groups based on the new model, cut-off points were determined by maximizing log rank statistic by minimizing the associated *p*-value. Visually, Kaplan-Meier survival curves (SPSS, SPSS Inc., Chicago, USA) were used to assess the risk stratification with respect to the OS of this patient cohort in comparison to previous prognostic indices. Harrell’s concordance index and log-rank statistics were used to compare the various signatures and models. The high and low risk groups identified were further analysed using PCA and hierarchical clustering in R and Qlucore Omics explorer.

## Results

### Patient characteristics

Serum samples from 44 out of 50 patients were available for the present analysis and collected from seven out of ten original clinical sites. Patients characteristics for the original and present cohort is presented in Table 1. The median follow-up time was 15 months with 18 deaths by the end of the study in 2018. The median age was 69 years with 73% of the patients above 65 years and 70% male patients. The median OS and PFS was about 15.0 and 13.7 months, respectively, similar to what has been previously reported by Owen et al*,* for relapsed MCL cohorts [30]. Among these 44 patients, three patients had missing information on MIPI and 12 lacked information on Ki-67. Within the MIPI distribution, 15% of the patients were low risk, 30% intermediate risk, and 50% high risk. More than 60% of the patients were attributed to high proliferation and Ki-67 was associated with increased risk (Table 1).

**Table 1:** Patient information

N (%)		Clinical trial Cohort	Present study
**Overall**		50 (100)	44 (100)
**Gender**	Male	36 (72)	31 (70.5)
	Female	14 (28)	13 (29.5)
**Age at diagnosis**	= < 65	15 (30)	14 (32)
	> 65	35 (70)	30 (68)
**MIPI**	Low risk	8 (16)	7 (16)
	Medium risk	15 (30)	13 (29.5)
	High risk	23 (46)	21 (47.7)
	Missing	4 (8)	3 (6.8)
**TP53**	Wild-type	23 (46)	20 (45.5)
	Deletions	17 (34)	14 (31.8)^a^
	Mutated	11 (25)	11 (25)^a^
	Missing	1 (2.3)	1 (2.3)
**Ki-67**	< 30%	17 (34)	12 (27)
	> 30%	21 (42)	20 (46)
	Missing	12 (24)	12 (27)
**Overall Survival (months)**	Median	15.08	15.08
	Number of deaths	20	18
**Time to progression (months)**	Median	13.65	13.65
	Number of deaths	24	21
**Median Overall Survival (months)**		15.08	15.08
**Median Time to Progression (months)**		13.65	13.65

*MIPI = Mantle Cell Lymphoma International Prognostic Index*

^a^*two patients had both TP53 deletion and mutation*

### Combined regression strategy, cox-BE and ENR, to identify proteins associated with OS

To identify proteins that were associated with OS, univariate cox regression and backward elimination (BE) were used. From the full microarray panel consisting of 371 analytes, the cox regression yielded 43 analytes with a *p*-value < 0.05 (Supplementary Fig. S3). All analytes were associated with improved outcome and had a hazard ratio (HR) < 1 with a range of 0.25–0.39 (Supplementary Fig. S3). Within this list of 43 analytes, there were 38 unique proteins, with PRD14, MCP-1, HER2/ERBB2, Eotaxin, Keratin19 having been identified by two different scFv’s.

To reduce the number of false positives, the 43 analytes underwent a second regression; stepwise backward elimination (see Materials and Methods for more detailed description). Overall, for each 12 runs of BE, an abridged version of condensed proteins was selected based on Wilcoxon *p*-value < 0.05 and was used for training and testing SVM leave-one-out cross-validation model. The average AUC across all runs was 0.67 with each run having an average of approximately 38 analytes. The 23 scFv’s that were identified in all 12 runs of BE were selected for further analysis (Supplementary Fig. S4). Among the 23 scFv’s, two clones were directed against MCP1. For further analysis, the MCP1 scFv with the superior p- value was selected. Thus, the final identified enriched panel as identified by Cox-BE contained 22 proteins.

To minimize false positive analytes, a secondary approach for regression was also utilised. Elastic net regression (ENR) was chosen as it is a relevant strategy when working with small and multicollinear datasets. Several ENR models (Materials and methods) were tested using the full microarray panel of 371 analytes. The optimal α = 0.5 was chosen based on lowest RMSE = 11.50 and the highest R^2^ = 0.64. This gave a list of 29 scFv’s and their respective ENR coefficients (Supplementary Fig. S5). The identified proteins IL-4, STAP2 and Factor B were represented by two clones each in the analysis. For two of the proteins (STAP2 and IL-4) the coefficient provided contradictory values. Both clones that identified Factor B showed a negative correlation (ENR coefficient for clone1 = − 1.99 and clone2 = − 0.54) in contrast to most other analytes with a positive correlation for OS. When multiple scFv’s were identified or for any contradictory results, the data from Cox regression was used. The ENR approached resulted in a protein panel of 26 proteins.

### Functional analysis and biological implication of the enriched protein panel

**Fig. 1:**
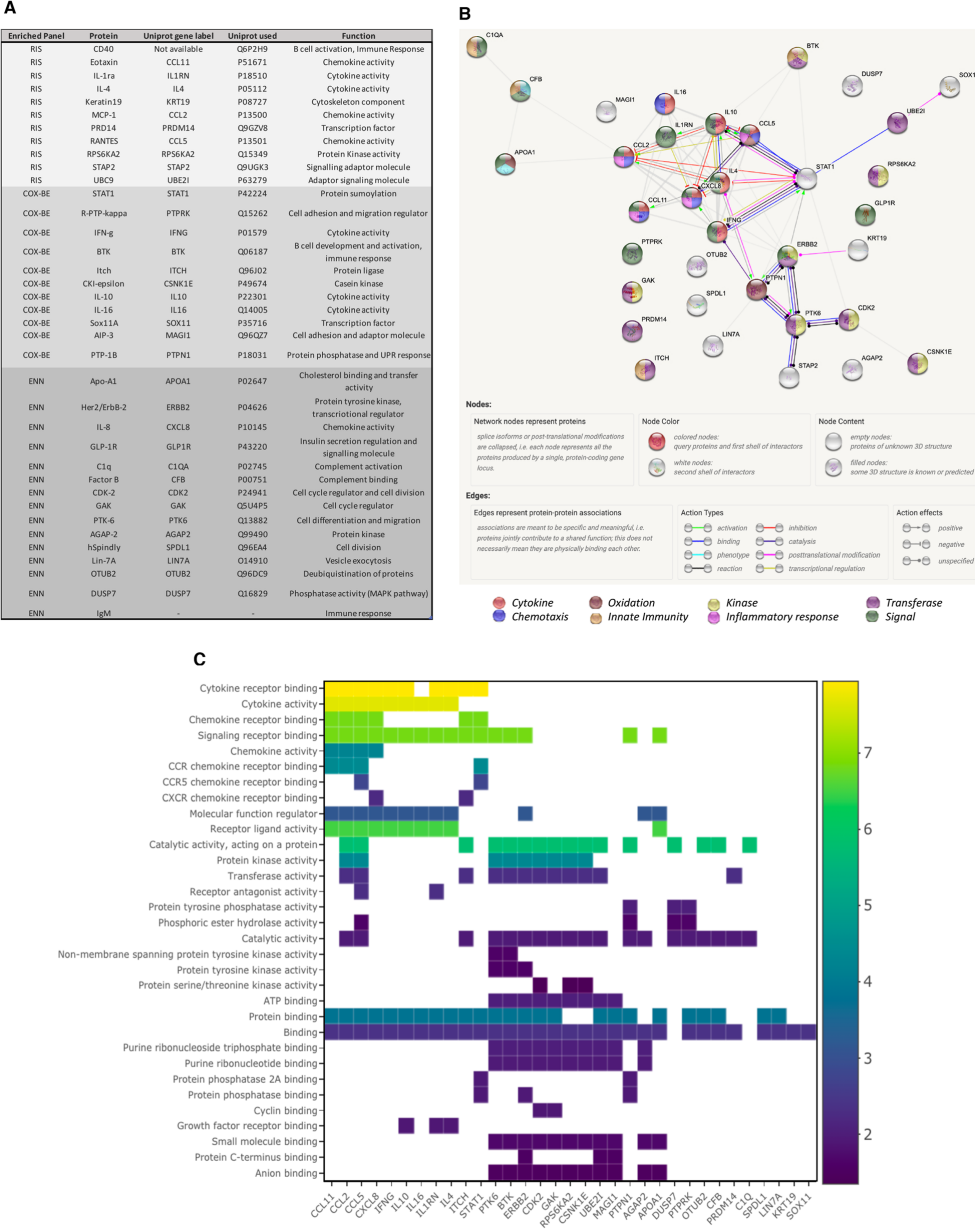
Overview and network analysis of identified serum proteins. **a** Cumulative protein list and their corresponding Uniprot gene label, Uniprot ID used for the pathway analysis; **b** Network map of all 35 proteins listed in (**a**) using String; **c**) Overall network profile of the molecular function plotted against enrichment score (log (1/*p*-value)), range: 1.88–7.94

From the two enriched panels identified above by Cox-BE and ENR, a total of 37 unique proteins were identified (Fig. 1a). To understand the multicollinearity within these 37 proteins and the possible biological interactions between them, a pathway analysis was performed. Figure 1a shows the 35 protein nodes (CD40 and IgM labels were unavailable). The results indicate that here is high degree of functional interaction between the different analytes. Of note, IL-4, IL-10, CCL2, CCL5, STAT-1 and IFNγ have a large degree of molecular interactions. The major functional activity of the proteins include cytokine and chemokine activity (Fig. 1c).

### Developing the relapsed MCL immune signature (RIS) and score based on selected proteins

The combined Cox-BE and ENR strategy identified 11 proteins that together stratify patients based on OS (Fig. 1a). Additional signature lengths were evaluated, but the overlapping signature of 11 proteins was validated to be the most significant using univariate cox analysis and Harrell’s concordance index.

For evaluating the efficacy of the signature in risk stratification, these 11 identified proteins were used to develop a relapsed MCL immune signature score (RIS score) as described in the Supplementary Materials and Methods. Univariate cox regression (OS, Table 2) was used to compare the efficacy of the RIS score to MIPI, Ki-67 (%), *TP53* mutational status or *TP53* deletion. It was shown that MIPI, RIS and Ki-67 were identified as significant factors associated with OS in this patient cohort (Table 2). However, the HR for Ki-67 barely reached significance at 1.023 (95% CI = 1–1.05, *P* < 0.05), probably due to limited number of patients. In comparison to MIPI with a HR of 1.97 (95% CI = 1.2–3.23, P < 0.05), RIS exhibited a 3.2 fold increase (95% CI = 1.33–7.77, *P* < 0.01), highlighting the strong association of the RIS score with OS. Gender was non-significant in both univariate and multivariate model and thus no impact on the significant variables mentioned above. Of note, univariate analysis of *TP53* mutation or deletion to OS was not significant in this patients cohort (Table 2) as reported previously [19], most likely due to the non-cytotoxic regiment used in the clinical trial protocol.

**Table 2:** Univariate cox regression analysis of RIS and previously defined prognostic factors

	n	β	HR (95% CI for HR)	P
**MIPI**	41	0.68	1.97 (1.2–3.23)	0.007
**RIS**	44	1.17	3.22 (1.33–7.77)	0.009
**KI67**	32	0.02	1.02 (1–1.05)	0.03
**Gender**	44	−0.12	0.89 (0.33–2.37)	0.81
***TP53***	43	0.46	1.58 (0.58–4.3)	0.37
**del** ***TP53***	43	−0.89	0.41 (0.13–1.33)	0.14

*HR* Hazard ratio, *β* risk coefficient, *P* p-value

### Comparison of the RIS to previous prognostic indices and development of the combined MCL relapsed immune signature index (MIPI_ris_)

To evaluate the impact of the developed immune-related score, RIS, together with MIPI, multivariate cox regression analysis was performed. The HR and β for RIS was 3.77 and 1.327 respectively (q < 0.01), nearly twice the impact compared to MIPI with HR = 2.03 and β = 0.708 (q < 0.01) (Table 3). Proliferation lost prognostic relevance in a multivariate model together with MIPI and RIS (Table 3), which may be related to that information on proliferation only was available for 30 patients which reduce statistical power. We additionally checked the performance of *TP53* mutation/deletion against MIPI and RIS in a multivariate analysis. However, it was non-significant and did not contribute towards the hazard risk. To evaluate the efficacy of a combined index taking both biological and patient-related parameters into account, we combined information from the 11 proteins constituting the RIS with the MIPI. The MIPI_ris_ was calculated as weighted sum of the MIPI and RIS and the weights were defined by the risk coefficients from the multivariate analysis (Supplementary Material and Methods). The new index was defined as: MIPI_ris_ = [0.708 X MIPI] + [1.327 X S11].

**Table 3:** Multivariate cox regression analysis to compare the significance of the RIS signature with respect to MIPI

		n	β	HR	Q
**A**	MIPI	41	0.708	2.03 (1.24–3.33)	0.009
	RIS	41	1.327	3.77 (1.38–10.3)	0.009
**B**	MIPI	30	0.778	2.18 (1.23–3.85)	0.023
	RIS	30	1.201	3.32 (1.18–9.34)	0.034
	Ki-67	30	0.017	1.02 (0.998–1.04)	0.081

*HR* Hazard ratio, *β* risk coefficient, *Q* q-value (FDR corrected p-value)

The median value of the MIPI_ris_ score was 4.87 (3.21–7.35). To trichotomize the data, potential cut-offs were evaluated by testing several iterations and final optimal boundary conditions were determined as 3.97 and 5.62 based on optimizing log-rank statistic and using minimal *p*-value approach (X^2^ = 12.883, *P* = 0.0016). Thus, the dataset was stratified in three subgroups as the following; low risk (LR) ≤ 3.97, 3.97 < intermediate risk (IR) < 5.62, 5.62 ≤ high risk (HR). The final patient distribution was nine patients (22%) in the low risk group, 29 patients (61%) in the intermediate risk group and six patients (17%) in the high risk group.

**Fig. 2:**
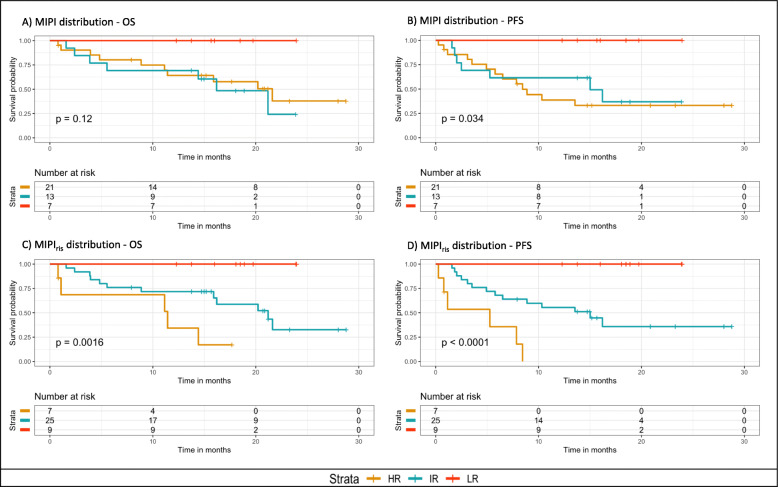
Kaplan Meier estimator comparing how MIPI and MIPI_ris_ stratify patients according to outcome. **a** and **b** are MIPI risk distribution (LR ≤ 5.7; 5.7 < IR < 6.2; 6.2 ≤ HR) with respect to overall survival and progression free survival respectively; **c** and **d** are MIPI_ris_ risk distribution (LR ≤ 3.97; 3.97 < IR < 5.62; 5.62 ≤ HR) with respect to overall survival and progression free survival respectively. LR = low risk, IR = intermediate risk, HR = high risk

MIPI alone failed to significantly divide the relapsed patients into distinct risk groups (log-rank statistic = 4.279, df = 2, *p* = 0.118), with the survival curves for high and the intermediate risk groups being undifferentiated and only low risk group separated (Fig. 2a). The patients were distributed over the different risk groups with 51.1% patients (*n* = 21) in the high risk group, 31.70% (*n* = 13) in the intermediate risk group and 17.01% (*n* = 7) in the low-risk group. In contrast, the combined MIPI_ris_, could clearly and significantly differentiate between the three subgroups with a log-rank *p*-value of 0.0016 (Fig. 2c). Also for progression free survival (PFS), the MIPI_ris_ had a stronger prognostic impact compared to MIPI alone (*p* < 0.0001 and *p* = 0.034, respectively). MIPI_ris_ could clearly separate the three risk groups with the survival curves for high and the intermediate risk groups clearly separated (Fig. 2c and d). Harrell’s concordance index performance was slightly better for MIPI_ris_ (0.714) compared to MIPI alone (0.662). Out of the seven patients in the low-risk MIPI group, six remained in the low-risk MIPI_ris_ group.

**Fig. 3:**
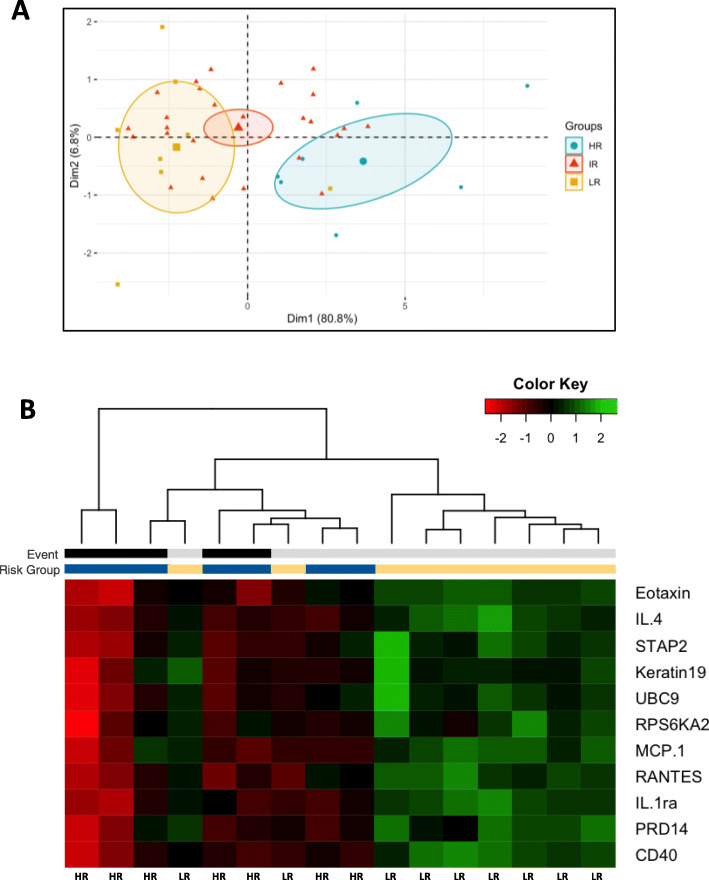
MIPI_ris_ risk distribution and variation within groups, highlighting high risk groups. **a** PCA risk distribution particularly differentiating high and low risk group, the ellipses represent the confidence of the distribution per risk category; **b** hierarchical clustering of the 11 antigens part of the relapsed MCL immune signature and MIPI_ris_; number of events (black: death, grey: live) and risk group (blue: high risk, yellow: low risk) is provided in the legend above

The overall risk distribution was visualized by PCA as shown in Fig. 3a, again exhibiting the segregation of high and low risk groups of the MIPI_ris_ by differential confidence clusters_._ The separation was primarily along the first component axis, likely explained by the overlapping functions of the individual RIS proteins as demonstrated by the pathway analysis. The expression of the 11 proteins in the defined RIS were increased in the low risk group compared to the high risk group (Fig. 3b).

## Discussion

Risk stratification is important for clinical practice, and the golden standard today is MIPI which was developed in 2008 for assessment of risk in newly diagnosed patients. The index is based on four parameters; age, LDH levels, WBC count and ECOG status [17]. However, MIPI does not take the biological heterogeneity into account, as limited molecular data is included [31]. Moreover, later studies where more modern treatment regimens are used, have shown that the index does not differentiate between low, intermediate and high risk groups of patients [32–35]. When combined with additional molecular information specific to individual patients, combined indices have improved risk stratification [31, 36–39]. The most widely used combined index is the MIPI-b where proliferation based on Ki-67 staining is added to the original MIPI [37]. Apart from this, there are several other prognostic factors and single markers identified in MCL such as the neural transcription factor *SOX11* [40] and several secondary mutations (*TP53, MYC, ATM, NOTCH1* etc.) [40]; among which *TP53* mutational status has shown largest impact on outcome [41]. However, there are limited studies on how soluble immune-related proteins in serum can contribute to assessment of risk, and stratification of MCL patients. To our knowledge, only a single study by Sonbol et al. has focused on cytokines in MCL blood samples [42], where sIL-2Rα, MIP-1β and IL-8 were identified as prognostic factors.

Thus, we have assessed the potential of using information in serum to associate combinations of proteins with response of relapsed MCL patients to treatment with immune-stimulatory agents. The identified signature, RIS, includes PRD14, STAP2, Eotaxin, RPS6KA2, Keratin19, MCP1, IL4, UBC9, IL1ra, RANTES and CD40. Most of the proteins have a known role in the immune system, but the majority have not been studied in the context of MCL biology or treatment response. Sonbol et al., identified IL1ra levels to be elevated in relapsed MCL in comparison to healthy controls, but was not correlated to event-free survival in that study [42]. Few of the RIS proteins have been studied in MCL tumor tissue, and thus we cannot speculate if the protein is leaking from the tumour cells, or altered as a response by the immune system to the tumor. However, RANTES (CCL5) has been reported to be overexpressed in MCL tumor tissue and MCL cell lines, tentatively playing a role in recruitment of T cells [43].

Several of the other proteins are well characterized in relation to their role in the immune system. IL-4 is a central hub for regulating immune behaviour and has previously been attributed to cell proliferation through the impact on CD40L [44]. CD40, a member of TNF receptor family plays an essential role in B cell proliferation, although it’s role in MCL has been under dispute [45–47]. While some studies suggest the involvement of CD40 in promoting MCL tumor cell proliferation, others debate the potential role in growth arrest [45, 47].

Of interest, the transcription factor PR domain zinc finger 14 (PRD14 or PRDM14), a regulator of pluripotency and epigenetic reprogramming in embryonic stem cells and germ cells [48–50], has not been studied in MCL, but other reports link high PRD14 to oncogenic behaviour in several cancer types including breast cancer and colorectal cancer [51–56]. It has been proposed that *PRDM14* (corresponding gene) expression could influence G1/S transition thus enabling cell proliferation [49] and facilitate cancer stem cell like properties and chemoresistance. Thus, inhibition of *PRDM14* has also been suggested as a potential target of treatment in cancer therapy [53]. Importantly, it has been shown that *PRDM14* overexpression leads to lymphoma formation in mice [57]. One of the other family members, *PRDM1*, is a master regulator of B-cell differentiation and acts as a tumor suppressor in DLBCL [58–60]. Potentially, it would be interesting to study *PRDM14* in MCL tissue to understand if it is expressed by the tumor cells or secreted by the immune system.

The first aim of the present study was to identify a signature that could stratify patients according to risk. This was assessed by calculating a patient-specific score based on the individual intensities weighted by their contribution in a combined multivariate analysis. The patient’s RIS score could then be used to stratify patients according to risk (OS). Additionally, we also evaluated the prognostic value of established risk factors, including proliferation and MIPI together with the RIS score. Both Ki-67 and MIPI were independently associated with OS in cox multivariate analysis together with RIS. However, RIS had a stronger impact with an increased HR of 3.3 as compared to 2.1 for MIPI and 1.02 for Ki-67. As reported previously, *TP53* aberrations were not correlated to OS in univariate analysis [19], potentially related to the fact that a non-chemotherapeutic regimen was used that do not depend on functional p53.

The second aim was to evaluate if risk stratification could further be improved by combining information from the RIS score with the clinically used MIPI. We show that MIPI alone does not significantly stratify these relapsed patients into distinct high, intermediate and low risk groups. When combining RIS and MIPI scores, and using optimized cut-offs, improved stratification was achieved. We show that MIPI_ris_ can improve separation between low, intermediate and high risk patients compared to MIPI alone, emphasizing that non-invasive sampling of immune-related serum proteins can be used to improve risk stratification in relapsed/refractory MCL patients. This newly defined index had a stronger impact than MIPI and MIPI-b in stratification using cox analysis, KM survival curves, log-rank statistics and Harrell’s concordance index.

Already today in the clinic, risk adapted therapy regimens based on MIPI are being explored [31]. In the diagnostic setting, low MIPI score patients are considered for wait-and-watch strategy whereas the intermediate or high risk group are proposed to be treated with combination chemotherapy (CHOP) and immune therapy (Rituximab), dosage depending on additional prognostic factors such as *TP53,* Ki-67 etc. [61]. Thus, improving stratification through addition of biological information can potentially enable better decision making for treatment regimens in both the diagnostic and relapsed setting.

The potential of including information on immune-related proteins is increasingly important for novel treatment strategies that often include immune stimulatory agents or strategies in both the diagnostic and relapsed setting. Most likely, the RIS protein panel is related to the specific treatment that the patients received, and the global applicability of such specific panels needs to be investigated in cohorts of patients receiving other treatment protocols.

## Conclusion

In this proof-of concept study, we have used three important concepts to risk-stratify patients, and enable improved clinical decision making through (i) minimally-invasive patient sampling, (ii) combined protein signature in contrast to single biomarkers and (iii) focus on immune-related information relevant to treatment outcome. We show that information from immune-related proteins in serum can be used alone or in combination with clinical parameters to improve stratification of patients treated with immune-stimulatory and targeted agents.

## Supplementary Information

**Additional file 1 Supplementary Table 1.** Antibody clones used in the microarray platform targeting a total of 158 unique antigens.

**Additional file 2 Supplementary Figure S1.** Example of an scanned array slide. The figure is an example of one scanned subarray slide, containing approximately ~ 190 different antibodies (half of the entire microarray set) printed as spots in triplicates. Each replicate set is bordered with the positive control (BSA-Biotin) and PBS was used as negative control.

**Additional file 3 Supplementary Figure S2.** An overview of the experimental and bioinformatic pipeline.

**Additional file 4 Supplementary Figure S3.** List of 43 analytes associated with overall survival based on univariate cox regression ordered as per their *p*-value significance. Black dots: Hazard ratio (HR) or exp.(β) where β is the risk coefficient, orange dots: 95% lower HR limit, blue dots: 95% higher HR limit. * Represents the marker identified by multiple scFv clones and the arrow marks indicate the duplicate clones; P: p-value.

**Additional file 5 Supplementary Figure S4.** Backward elimination coupled with SVM and LOOCV post cox regression. A) Boxplot of the ROC-AUC values across all 12 iterations of BE; and B) the frequency of appearance of each 43 analyte as identified previously by cox regression. In total, 23 analytes for 22 unique serum proteins (MCP1 identified by 2 scFv’s) were identified in all 12/12 iterations (as highlighted by the red box) which were selected as the enriched panel for Cox-BE.

**Additional file 6 Supplementary Figure S5.** Illustration of the second regression approach using elastic net regression (ENR). The entire microarray panel of 371 analytes were used. A) error graph for the log (lambda) values. B) Coefficient collapse to zero for α = 0.5, wherein only 29 analytes were eventually selected with non-zero coefficients. C) The regressed panel of 29 parameters plotted against the ENR coefficients. Coefficient < 0 (“Negative”) implies negative correlation to OS; Coefficient > 0 (“Positive”) implies positive correlation to OS.

**Additional file 7.** Supplementary materials and methods.

## Data Availability

The datasets used and/or analysed during the current study are available from the corresponding author on reasonable request. Supplementary figures (1, 2, 3, 4 and 5) and Table 1, as well as supplementary materials and methods are provided.

## Abbreviations

MCL: Mantle Cell Lymphoma
OS: Overall Survival
PFS: Progression Free Survival
MIPI: Mantle cell lymphoma International Prognostic Index
MIPI_ris_: Mantle cell lymphoma International Prognostic Index Relapsed Immune Signature
RIS: Relapsed Immune Signature
