# Supplementary figures and images for: Immune-related protein signature in serum stratify relapsed mantle cell lymphoma patients based on risk
Source: BMC Cancer. 2020 Dec 7;20:1202. doi: 10.1186/s12885-020-07678-4 (PMC7720632; doi:10.1186/s12885-020-07678-4)

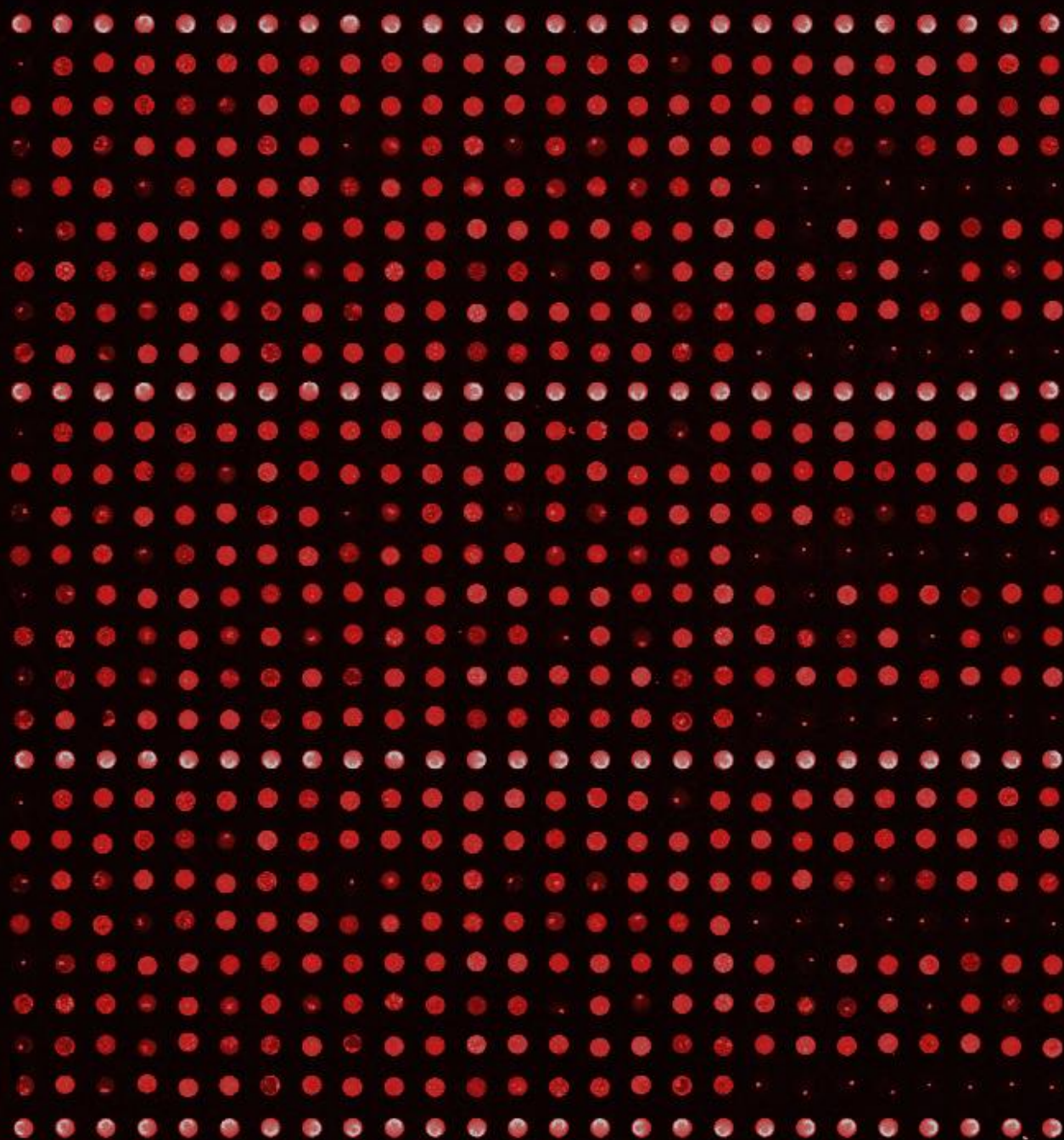

Supplement: Supplementary file 2 — Additional file 2 Supplementary Figure S1. Example of an scanned array slide. The figure is an example of one scanned subarray slide, containing approximately ~ 190 different antibodies (half of the entire microarray set) printed as spots in triplicates. Each replicate set is bordered with the positive control (BSA-Biotin) and PBS was used as negative control. [file 12885_2020_7678_MOESM2_ESM.pdf]

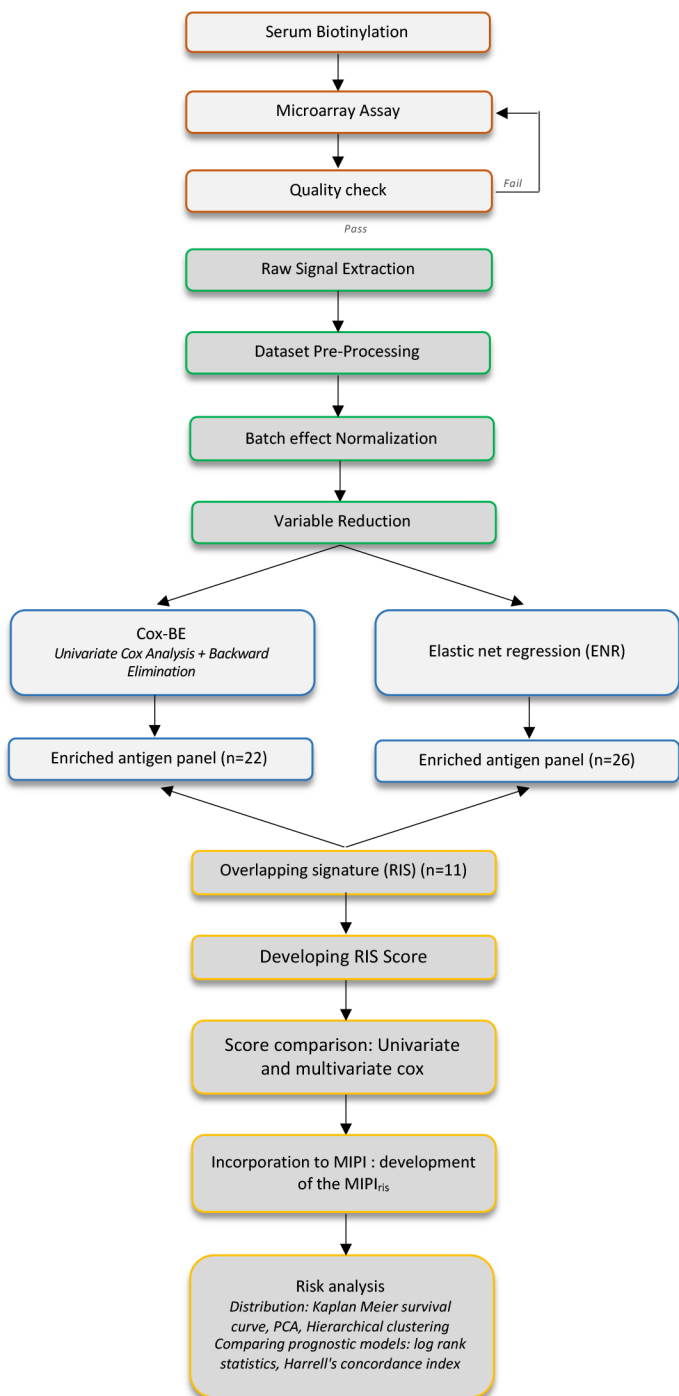

Supplement: Supplementary file 3 — Additional file 3 Supplementary Figure S2. An overview of the experimental and bioinformatic pipeline. [file 12885_2020_7678_MOESM3_ESM.pdf]

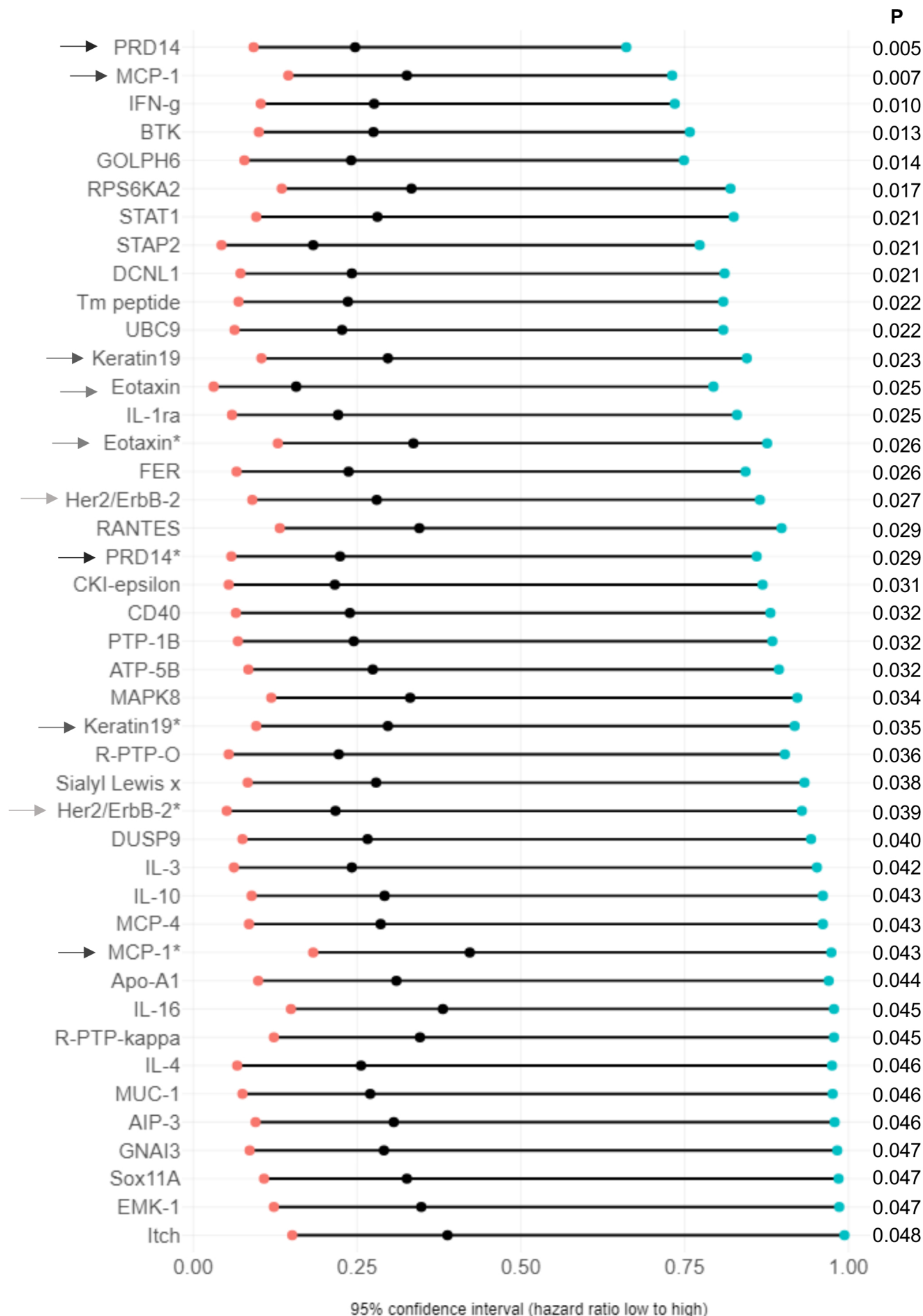

Supplement: Supplementary file 4 — Additional file 4 Supplementary Figure S3. List of 43 analytes associated with overall survival based on univariate cox regression ordered as per their p-value significance. Black dots: Hazard ratio (HR) or exp.(β) where β is the risk coefficient, orange dots: 95% lower HR limit, blue dots: 95% higher HR limit. * Represents the marker identified by multiple scFv clones and the arrow marks indicate the duplicate clones; P: p-value. [file 12885_2020_7678_MOESM4_ESM.pdf]

A

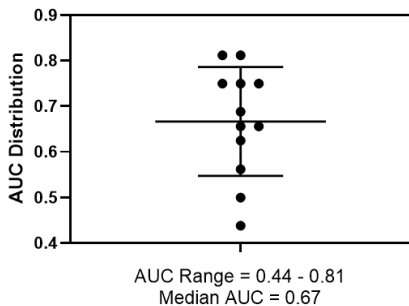

B

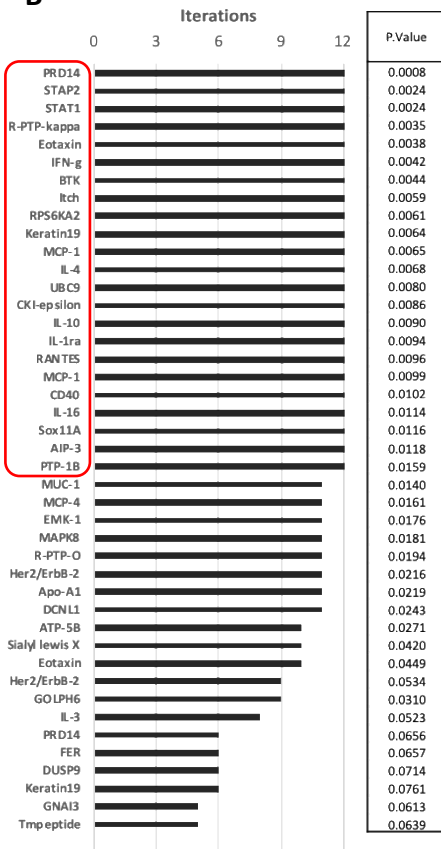

Supplement: Supplementary file 5 — Additional file 5 Supplementary Figure S4. Backward elimination coupled with SVM and LOOCV post cox regression. A) Boxplot of the ROC-AUC values across all 12 iterations of BE; and B) the frequency of appearance of each 43 analyte as identified previously by cox regression. In total, 23 analytes for 22 unique serum proteins (MCP1 identified by 2 scFv’s) were identified in all 12/12 iterations (as highlighted by the red box) which were selected as the enriched panel for Cox-BE. [file 12885_2020_7678_MOESM5_ESM.pdf]

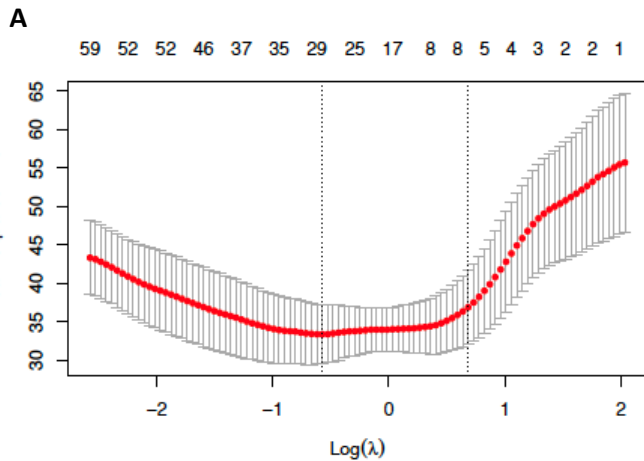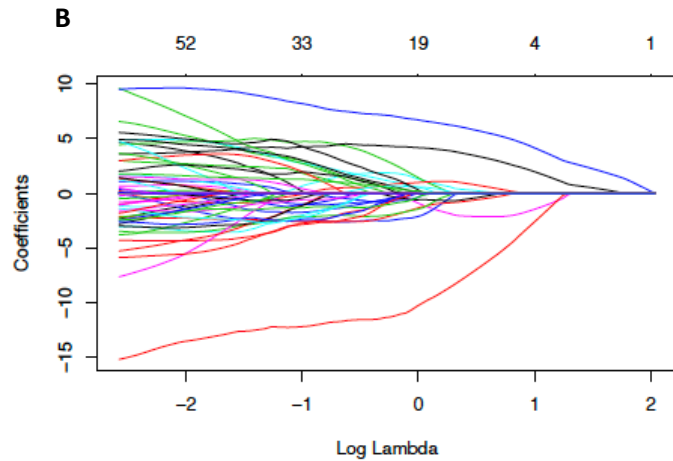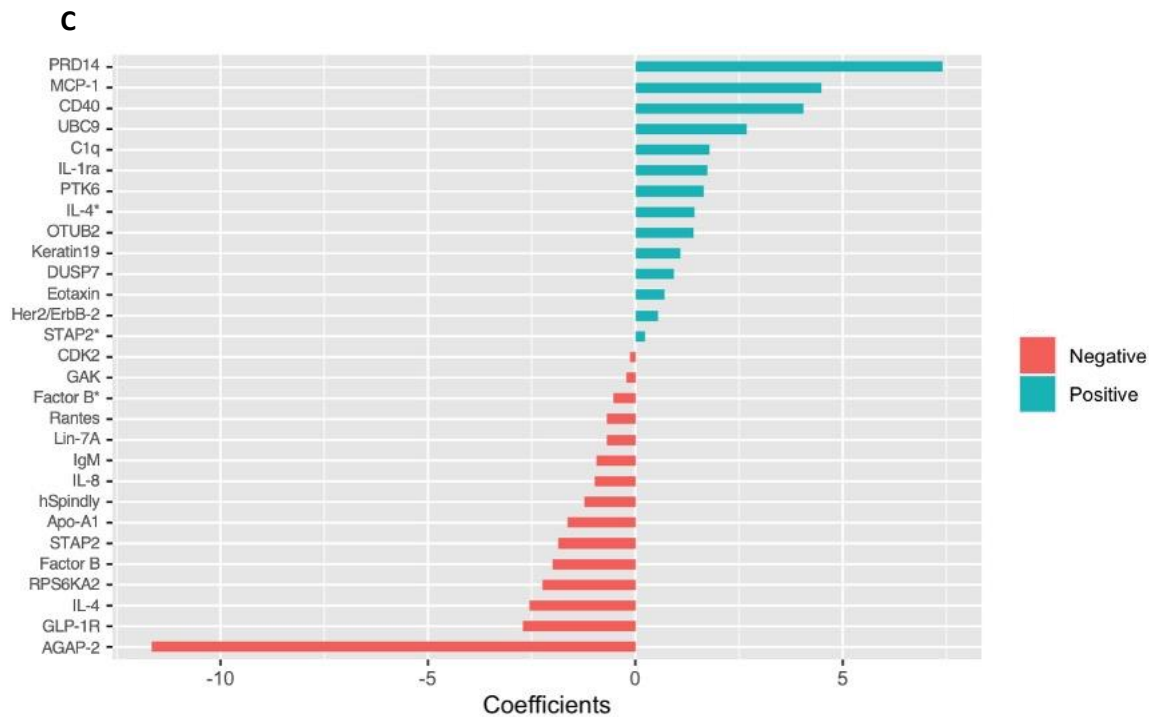

Supplement: Supplementary file 6 — Additional file 6 Supplementary Figure S5. Illustration of the second regression approach using elastic net regression (ENR). The entire microarray panel of 371 analytes were used. A) error graph for the log (lambda) values. B) Coefficient collapse to zero for α = 0.5, wherein only 29 analytes were eventually selected with non-zero coefficients. C) The regressed panel of 29 parameters plotted against the ENR coefficients. Coefficient < 0 (“Negative”) implies negative correlation to OS; Coefficient > 0 (“Positive”) implies positive correlation to OS. [file 12885_2020_7678_MOESM6_ESM.pdf]
