# Supplementary material for: Immune-related protein signature in serum stratify relapsed mantle cell lymphoma patients based on risk
Source: BMC Cancer. 2020 Dec 7;20:1202. doi: 10.1186/s12885-020-07678-4 (PMC7720632; doi:10.1186/s12885-020-07678-4)
Supplement: Supplementary file 7 — Additional file 7. Supplementary materials and methods. [file 12885_2020_7678_MOESM7_ESM.pdf]

Supplementary Materials and Methods

S1. Regression using stepwise backward elimination

The backward elimination is combined with a support vector machine (SVM). Briefly, the dataset was randomly split in training (70%) and test/validation (30%) groups. Missing values (if any) in the dataset were bag imputed prior to the backward elimination runs. In total, twelve backward elimination runs were performed, with five (n=6) or two (n=6) parallelized outer loops, ten repeats of cross-validation in each run,using the receiving operating characteristics (ROC) as the classification error metric for optimization. After each run, all antibodies were ranked based on the Wilcoxon p-values and the scFv's with p<0.05 were selected for cross-validation and represented the analyte signature. A frozen SVM was trained for each run using the training dataset across each outer fold and further validated using leave-one-out cross validation on the test set, which results in a ROC curve and a cumulative AUC value for each consolidated BE-SVM run. The average AUC across all runs was 0.67 with each run having an average signature length of approximately 38 analytes. Finally, a consensus approach was used to find a condensed antigen panel, wherein the number of occurrences for each analyte was counted and final panel was selected based on the maximum number of events.

S2. Actual Model Calculation

The RIS score (SS) was calculated as –

$$SS = \text{Avg}(\beta_i S_i) - Median(\text{Avg}(\beta_i S_i))$$

β_i = Coefficient from Univariate Cox analysis for each serum biomarker "*i*" constituting the final signature of length 11.
S_i = Normalized signal intensity from the microarray data for each serum biomarker "*i*" constituting the final signature of length 11.
The median value was subtracted for scaling the overall score, which also resulted in continuous, but partially categorical, ternary division of the dataset. Therefore, the final scaled signature score was in the range of: -1.05 to 1.56.

The MIPI_{ris} score was calculated as –

$$\text{MIPI}_{ris} = 0.708(MIPI) + 1.327(SS)$$

Optimal cut-off thresholds for MIPI_{ris} were defined by testing several iterations to find out the maximized log rank value and minimizing the p-value [17]. Therefore, a linear predictive model against overall survival for this dataset was generated (n=44); with a continuous score and categorical risk division was developed.
